# Insecticidal features displayed by the beneficial rhizobacterium *Pseudomonas chlororaphis* PCL1606

**DOI:** 10.1007/s10123-022-00253-w

**Published:** 2022-06-07

**Authors:** Eva Arrebola, Francesca R. Aprile, Claudia E. Calderón, Antonio de Vicente, Francisco M. Cazorla

**Affiliations:** 1 Departamento de Microbiología, Facultad de Ciencias, Universidad de Málaga, Málaga, Spain; 2 Instituto de Hortofruticultura Subtropical y Mediterránea “La Mayora”, Consejo Superior de Investigaciones Científicas, Universidad de Málaga IHSM-UMA-CSIC, Málaga, Spain

**Keywords:** Insecticidal, *Galleria mellonella*, *Pseudomonas chlororaphis*, Avocado, Biocontrol

## Abstract

The biocontrol rhizobacterium *Pseudomonas chlororaphis* is one of the bacterial species of the *P. fluorescens* group where insecticide *fit* genes have been found. Fit toxin, supported with other antimicrobial compounds, gives the bacterial the ability to repel and to fight against eukaryotic organisms, such as nematodes and insect larvae, thus protecting the plant host and itself. *Pseudomonas chlororaphis* PCL1606 is an antagonistic rhizobacterium isolated from avocado roots and show efficient biocontrol against fungal soil-borne disease. The main antimicrobial compound produced by *P. chlororaphis* PCL606 is 2-hexyl-5-propyl resorcinol (HPR), which plays a crucial role in effective biocontrol against fungal pathogens. Further analysis of the *P. chlororaphis* PCL1606 genome showed the presence of hydrogen cyanide (HCN), pyrrolnitrin (PRN), and homologous *fit* genes. To test the insecticidal activity and to determine the bases for such activity, single and double mutants on the biosynthetic genes of these four compounds were tested in a *Galleria mellonella* larval model using inoculation by injection. The results revealed that Fit toxin and HPR in combination are involved in the insecticide phenotype of *P. chlororaphis* PCL1606, and additional compounds such as HCN and PRN could be considered supporting compounds.

**Supplementary Information:**

The online version contains supplementary material available at 10.1007/s10123-022-00253-w.

## Introduction

A wide variety of microorganisms are living in the soil, and they are concentrated around the roots of plants. Beneficial microorganisms coexist in this ecological niche along with pathogens and predators (Vesga et al. 2021). Among the beneficial rhizobacteria associated with plant roots, the genus *Pseudomonas* is one of the most studied. The principal interactions of these rhizobacteria with the plants include cooperation with the plant host by plant growth promotion (PGPR), induction of systemic acquired resistance (SAR; Shifa et al. 2018; Kamle et al. 2020), and competition or antagonism to soil-borne phytopathogens. In addition, these bacteria can show insecticidal activity and use insects as vectors for dispersal (Esposti and Romero 2017; Flury et al. 2019).

The main group PGPR pseudomonads is the *Pseudomonas fluorescens* group, where insecticidal activity is not a common trait. Moreover, according to genomic diversity analysis, the *P. fluorescens* group is divided into three subclades (Loper et al. 2012; Flury et al. 2016). Interestingly, the strains belonging to subclade number 2 neither harbor the Fit toxin nor have the ability to kill *Galleria mellonella* larvae (Ruffner et al. 2015). However, all strains belonging to *P. protegens* and *P. chlororaphis* species tested in that study (which represent subclade 1) have both entomotoxicities (Ruffner et al. 2015; Flury et al. 2016). There are some examples of *P. protegens* and *P. chlororaphis* strains that are able to infect and efficiently kill insect larvae after oral uptake (Kupferschmied et al. 2013; Flury et al. 2016; Vesga et al. 2020), and this trait could also be related to the rhizobacterial ability to resist the deleterious effects of grazing predators, resulting in better host protection and competition against surrounding organisms (Nandi et al. 2015; Schellenberges et al. 2016).

A close association with insecticidal activity has been demonstrated in the model bacterium *P. protegens* Pf-5 for a set of genes named the *fit*ABCDEFGH cluster (*fit*, fluorescens insecticidal toxin; Péchy-Tarr et al. 2008, 2013; Ruffner et al. 2015). The unique virulence cassette harbors the *fit*D gene encoding the Fit toxin as well as regulatory and secretion protein functions (Péchy-Tarr et al. 2008). However, *fit*D deletion mutants retain substantial toxicity, indicating the presence of additional virulence factors (Péchy-Tarr et al. 2008; Ruffner et al. 2015). Many antagonistic pseudomonads are able to produce several antimicrobial compounds, such as phenazines (PHZ), 2,4-diacetylphloroglucinol (DAPG), pyoluteorin (PLT), pyrrolnitrin (PRN), hydrogen cyanide (HCN), or cyclic lipopeptides (Jang et al. 2013; Flury et al. 2017), that form a cocktail able to repel plant pathogens (Haas and Keel 2003; Gross and Loper 2009; Rochat et al. 2010). It has been reported that some of these primary antifungal compounds can also be used for self-defense against predators, such as protozoa and nematodes (Bjørnlund et al. 2009; Jousset et al. 2009; Raaijmakers and Mazzola 2012). Specifically, HCN has shown nematicidal activity against *Meloidogyne hapla*, supported by PHZ and PRN production (Lee and Ma 2011). Moreover, Nandi et al. (2015) reported that HCN and PRN were able to act as powerful fungal repellents; in addition, these two compounds are the key compounds that affect the interaction of *P. chlororaphis* PA23 and *Caenorhabditis elegans*. In this sense, *Galleria mellonella* larvae are being used as an insect model for antibiotic susceptibility testing (Tsai et al. 2016; Ignasiak and Maxwell 2017; Andrea et al. 2019), toxicity of chemicals (Allegra et al. 2018), and virulence factors (Durieux et al. 2021), revealing their usefulness for insecticidal activity studies of several rhizobacteria.

The model rhizobacterium *P. chlororaphis* PCL1606 was isolated from the root of healthy avocado trees (*Persea americana* Mill.) in a crop infected by *Rosellinia necatrix*, the causal agent of white rot root. This bacterium is characterized by being a highly efficient antagonist against many soil-borne phytopathogenic fungi (Cazorla et al. 2006; Calderón et al. 2014). Analysis of antifungal compounds produced by PCL1606 has shown the production of HCN and PRN; however, its main antifungal antibiotic produced by PCL1606 is 2-hexyl-5-propyl resorcinol (HPR; Cazorla et al. 2006; Calderón et al. 2013). Another characteristic of this unusual strain is its disability to produce phenazine, a representative antibiotic of the specie *Pseudomonas chlororaphis* (Calderón et al. 2015).

Previous studies using random insertional derivative Tn*5* mutants and selected insertional defective mutants lacking HPR have indicated a direct correlation between resorcinol and the biocontrol of soil-borne fungal diseases (Cazorla et al. 2006; Calderón et al. 2013). Furthermore, the locus encoding a putative cytotoxin homologous to FitD has been allocated to the PCL1606 genome (Calderón et al. 2015). These circumstances suggest that *P. chlororaphis* PCL1606 could be a good candidate to have insecticidal functions.

The main goal of this study was to unravel the roles of HCN, PRN, HPR, and FitD compounds in the insecticidal features of *P. chlororaphis* PCL1606. For this, the insecticidal activity of PCL1606 was tested in a *Galleria mellonella* (greater wax moth) model. Although this model has no agricultural interest, it is very useful to make a first approximation in the insecticidal abilities of PCL1606.

## Materials and methods

### Bacterial strains, plasmids, and growth conditions

The plasmids and bacterial strains are described in Table 1. *Escherichia coli* strains were grown on LB medium (Ausubel et al. 1995) at 37 °C for 24 h and supplemented with antibiotics according to the plasmid requirements. *Pseudomonas* spp. were grown and maintained on LB broth, KB medium (King et al. 1954), and/or tryptone-peptone-glycerol medium (TPG, Calderón et al. 2013), supplemented with antibiotics as necessary (Table 1), and incubated at 25 °C for 48 h. The antibiotic concentrations used in this study were 50 µg/mL kanamycin (km), 80 µg/mL gentamycin (Gm) for defective *P. chlororaphis* PCL1606 mutants, and 40 µg/mL for the *E. coli* strain. *Galleria mellonella* larvae were obtained by commercial production (Animal-Center S.C., Spain), used immediately and maintained at 25 °C in the dark during the experiments, and eventually preserved for 1 week at 4–10 °C.

**Table 1:** Bacteria and plasmids used in the current study

Strain	Relevant characteristics^a^	Reference
Bacteria		
* Pseudomonas* spp.		
AVO110	*P. alcaligenes*; efficient colonizer of avocado roots; antagonistic to *Rosellinia necatrix*	Pliego et al. 2007; Pintado et al. 2021
BL915	*P. chlororaphis* subsp. aurantiaca; antagonistic to *Rhizoctonia solani*	Hill et al. 1994; Nowak-Thompson et al. 2003
Pf-5	*P. protegens*; antagonistic to *Rhizoctonia solani*; insecticidal activity on *Galleria mellonella*	Howell and Stipanovic 1979; Péchy-Tarr et al. 2008; Lim et al. 2013
PCL1606	*P. chlororaphis*; isolated from avocado rhizosphere; biocontrol, efficient root colonizer and antagonistic to *Rosellinia necatrix* and *Fusarium oxysporum*	Cazorla et al. 2006
PCL1606::*dar*B	PCL1606 derivative insertional mutant in *dar*B gene; HPR-; Km^r^ (former name Δ*dar*B)	Calderón et al. 2013, 2019
ComB	PCL1606::*dar*B transformed with pCOMB; HPR + ; Gm^r^	Calderón et al. 2013
PCL1606::*prn*C	PCL1606 derivative insertional mutant in *prn*C gene; PRN-	Calderón et al. 2015
PCL1606::*hcn*B	PCL1606 derivative insertional mutant in *hcn*B gene; HCN-	Calderón et al. 2015
PCL1606::*fit*D	PCL1606 derivative insertional mutant in locus PCL1606_RS12180	This study
PCL1606::*dar*B*prn*C	PCL1606 derivative double insertional mutant in *dar*B and *prn*C genes; HPR-; PRN-	Calderón et al. 2015
PCL1606::*dar*B*hcn*B	PCL1606 derivative double insertional mutant in *dar*B and *hcn*B genes; HPR-; HCN-	Calderón et al. 2015
PCL1606::*prn*C*hcn*B	PCL1606 derivative double insertional mutant in *prn*C and *hcn*B genes; PRN-; HCN-	Calderón et al. 2015
PCL1606::*dar*B*fit*D	PCL1606 derivative double insertional mutant in *dar*B and *fit*D genes; HPR-; FIT-	This study
PCL1606::*gac*S	PCL1606 derivative insertional mutant in *gac*S gene, involved in secondary metabolism regulation	Martín-Pérez et al. 2007
* Escherichia coli*		
DH5α	General cloning and sub-cloning applications; dlacZ Delta M15 Delta(lacZYA-argF) U169 recA1 endA1 hsdR17(rK-mK +) supE44 thi-1 gyrA96 relA1	Taylor et al. 1993
Plasmids		
pCR®2.1-TOPO®	PCR products cloning vector *lacZ*, Km^r^, Amp^r^	Invitrogen, California, USA
pJQ200SK	Suicide vector, P15A *ori*V *sac*B *mob*, Gm^r^	Quandt and Hynes 1993
pCRfitD	A fragment of PCL1606_RS12180 [PCL1606_24850] sequence cloned into pCR2.1, Km^r^ for integrative mutation	This study
pJQfitD	A fragment of PCL1606_RS12180 [PCL1606_24850] sequence cloned into pJQ200SK, Km^r^ for integrative mutation in PCL1606::*darB*	This study

^a^HCN, production of hydrogen cyanide; HPR, production of 2-hexyl-5-propyl resorcinol; PRN, production of pyrrolnitrin; FIT, production of FitD protein; Km^r^, kanamycin resistant; Gm^r^, gentamycin resistant; Amp.^r^, ampicillin resistant

### Insecticidal activity in Galleria mellonella

The insecticide capacity of *Pseudomonas* spp. strains was performed in a *Galleria mellonella* larval model (Burges 1976). The *Pseudomonas* spp. strains were grown in 30 mL of LB broth without antibiotics at 25 °C overnight and 200 rpm of orbital agitation. Afterwards, the cultures were adjusted to 3 × 10^5^ cfu/mL or 3 × 10^3^ cfu/mL according to the assay. Ten milliliters from cultures was centrifuged at 4000 rpm for 10 min, and the pellets were resuspended in the same volume using 10 mM MgSO_4_ buffer.

Commercial *Galleria mellonella* larvae approximately 1.5-cm long and 0.5-cm wide in 6th larval stage were selected for assays. The larvae were inoculated with approximately 10 µL of bacterial suspension injected into a 1-mL syringe with a needle 13-mm long and a 0.3-mm internal diameter (Becton Dickinson, Ireland). The needle and inoculation point were disinfected by 96% ethanol every time. Injections with strain Pf-5 (Table 1) and MgSO_4_ buffer were performed as positive and negative controls, respectively.

The experiments were performed by three independent assays with 15 larvae each. Once inoculated, the larvae were kept in the dark at 25 °C for 3.5 days. Insecticidal activity was monitored after 17, 24, 30, 40, 60, and 80 h by checking the absence of motility and melanization of the larval body.

### Strain manipulation and molecular assays

Insertion mutagenesis for gene inactivation in *P. chlororaphis* PCL1606 was used to test the insecticide characteristics. Specifically, for this study, a disruptive vector was inserted into the putative *fit*D gene located on the PCL1606 chromosome via single-crossover homologous recombination (Arrebola et al. 2012). The cloning of vector pCR2.1 (Invitrogen Life Technologies USA) and plasmid purification were performed using standard procedures. The plasmids obtained were transformed into wild-type PCL1606 by electroporation (Choi et al. 2006). The double insertional mutant PCL1606::*dar*B*fit*D was constructed using the previously obtained single insertional mutant PCL1606::*dar*B (Calderón et al. 2013) in which the pCR2.1 derivative plasmid was already present in the mutant. Consequently, the suicide vector pJQ200SK (Table 1) was used to clone a *fit*D gene fragment and mutate the *fit*D gene by insertion of the chromosome via single-crossover homologous recombination.

The correct insertions of the disruption vectors were verified by PCR amplification. Bacterial growth curves were obtained in LB broth culture media to confirm similarities among the constructed defective mutants and the wild-type *P. chlororaphis* PCL1606 (data not shown).

### Statistical analysis

The data were statistically analyzed using an analysis of variance ANOVA (Sokal and Rohlf 1986), followed by Fisher’s least significant difference test (LSD, *P* = 0.05) using IBM SPSS Statistics 22 software (SPSS Inc., Chicago, IL, USA). All experiments were performed at least three times independently. Survival of infected larvae (*n* = 20 per group) following treatment was recorded every 24 h for 96 h. Larvae were considered dead when they failed to respond to touch. The control groups were infected larvae treated with 10 μl of PBS-T. Kaplan–Meier survival curves were plotted using data pooled from a minimum of two independent experiments.

## Results

### Genotypic and phenotypic study of the insecticidal capacity of P. chlororaphis PCL1606

**Fig. 1:**
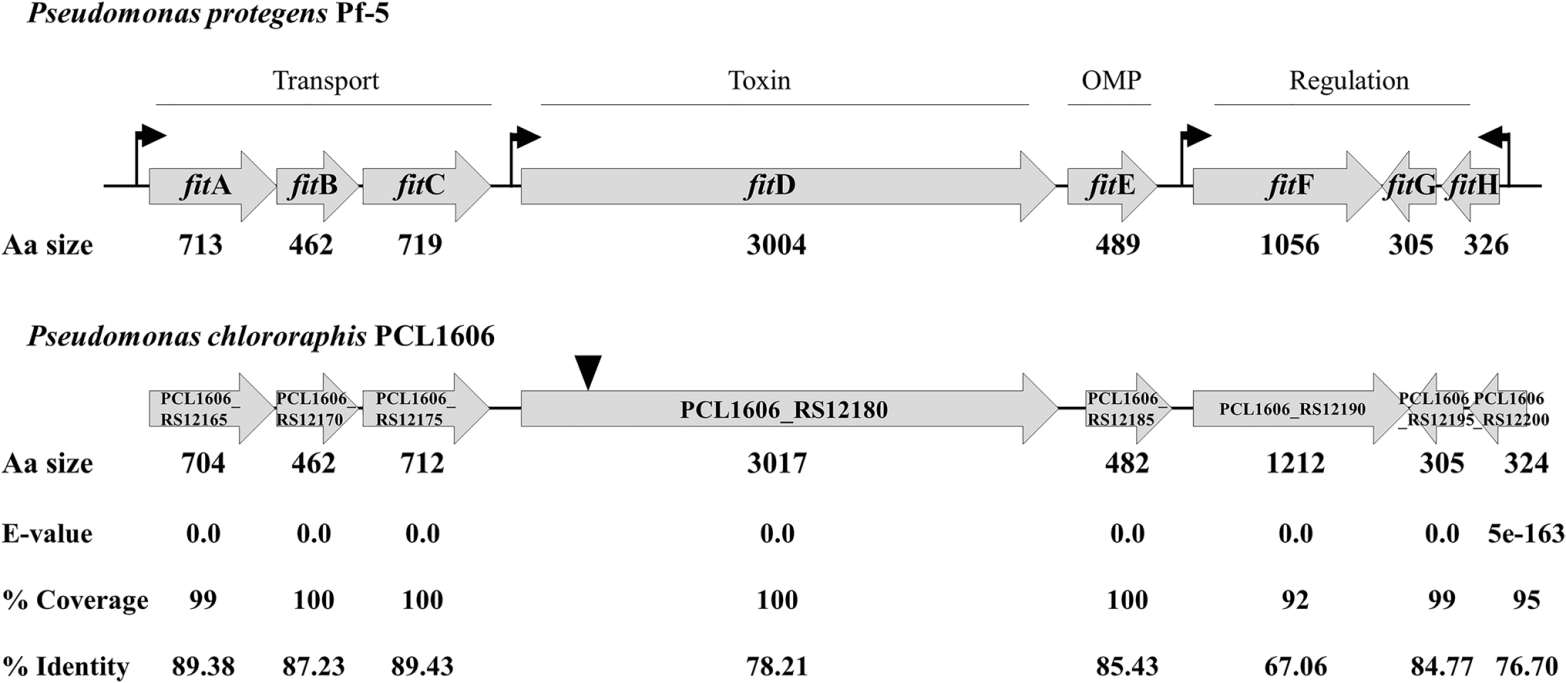
Organization of the insect toxin cluster in *Pseudomonas protegens* Pf-5, based on Péchy-Tarr et al. (2008) information, and *Pseudomonas chlororaphis* PCL1606. Protein sizes are indicated as number of amino acid (Aa size) for *P. protegens* Pf-5 and *P. chlororaphis* PCL1606. Besides, reliability of comparison (*E* value), percentage of unique reads (% coverage), and percentage of nucleotides identical (% identity) indicate for *P. chlororaphis* PCL1606. The mutation point is indicated by black inverted triangle. Genes with transport function, outer membrane protein (OMP), toxin, and regulation putative functions are also indicated

An in silico analysis of putative *fit* genes found in *P. chlororaphis* PCL1606 revealed a high similarity with *fit* gene operons detected in the insecticidal bacterium *Pseudomonas protegens* Pf-5, in which putative products are equivalents (Fig. 1). Loci PCL1606_RS12165, PCL1606_RS12170, and PCL1606_RS12175 are related to the transport of cytotoxin, PCL1606_RS12180 is the largest locus and corresponds to the insect toxin FitD, PCL1606_12185 is annotated as an outer membrane protein, and PCL1606_RS12190, PCL1606_RS12195, and PCL1606_RS12200 are related to the regulation of toxin production (Table 2). Equivalent genes were described in *P. protegens* Pf-5 and CHA0, where *fit*ABC is related to transport by the type I secretion system, *fit*D has been described as a cytotoxin coding gene, *fit*E as a type I secretion outer membrane protein. and *fit*FGH as a regulatory gene (Péchy-Tarr et al. 2008). Furthermore, the distribution and arrangement of these loci in the genome of PCL1606 are homologous to the *fit* cluster in *P. protegens* Pf-5. Likewise, the protein size and percentage of identity of the majority of loci with its equivalent *fit* gene showed that it was highly similar (Fig. 1). Once the putative *fit*D gene was located in PCL1606, an insertional defective mutant was constructed to analyze its involvement in insecticidal activity. Commercial *Galleria mellonella* larvae were used as model of insecticidal activity of *P. chlororaphis* PCL1606 derivative mutant in antibiotic compounds, such as pyrrolnitrin (PRN), hydrogen cyanide (HCN), and 2-hexyl-5-propyl resorcinol (HPR). Besides, the mutant defective in the global regulator GacS was also studied (Table 1).

**Table 2:** *Pseudomonas chlororaphis* PCL1606 gene description used in the current study and tested by directed mutagenesis. Information obtained from the National Center for Biotechnology Information (NCBI)

Locus	Gene	Product description	% Identity ncl-ncl	Reference Microorganism
PCL1606_RS08425	*gac*S	Response regulator	94	*Pseudomonas chlororaphis* 189
PCL1606_RS10175	*dar*B	β-ketoacyl synthase DarB	92	*Pseudomonas aurantiaca* BL915
PCL1606_RS12180	*fit*D	Cytotoxin	84	*Pseudomonas protegens* Pf-5
PCL1606_RS13730	*prn*C	FAD-dependent oxidoreductase	94	*Pseudomonas aurantiaca* BL915
PCL1606_RS17610	*hcn*B	Cyanide-forming glycine dehydrolase subunit HcnB	85	*Pseudomonas protegens* CHA0

**Fig. 2:**
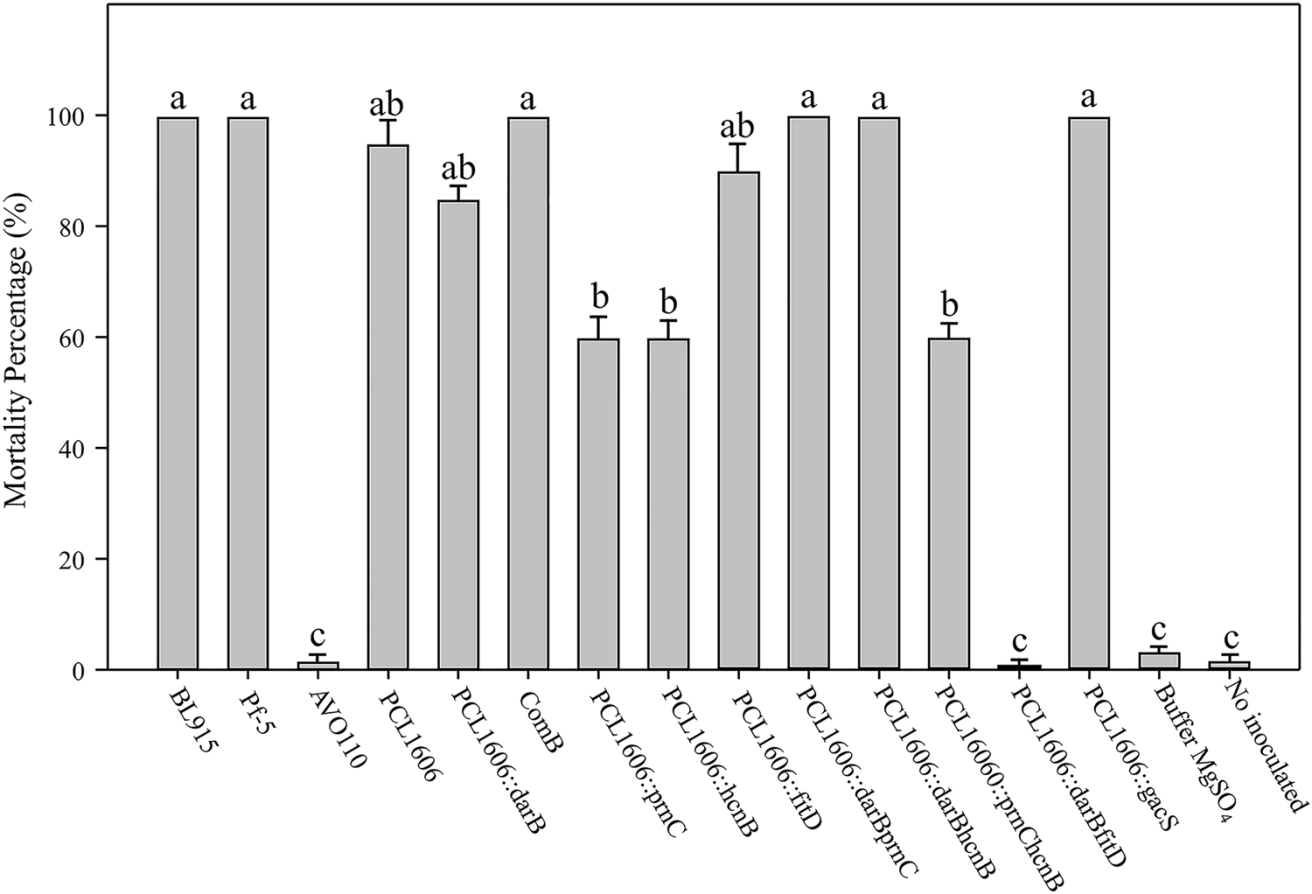
Mortality percentage of *Galleria mellonella* larvae, infected by injection of dose 3 × 10.^5^ cfu/mL and 24 h of incubation at 25 °C. Statistical analysis by ANOVA was performed using IBM SPSS 22 software (SPSS Inc., Chicago, IL, USA) and error bars are represented

The results have been compared with the wild-type strains *P. protegens* Pf-5, where a *fit* cluster was described (Péchy-Tarr et al. 2008), *P. chlororaphis* subsp*. aurantiaca* BL915, a producer of HPR (Nowak-Thompson et al. 2003), and *P. alcaligenes* AVO110, whose biocontrol activity was not related to antibiotic production (Pliego et al. 2007; Pintado et al. 2021). Twenty-four hours postinoculation with 3 × 10^5^ cfu/mL as a dose (Fig. 2), the control wild-type strains Pf-5, BL915, and PCL1606 caused a high insect mortality percentage (100% of dead larvae with intense larval melanization), contrary to AVO110, in which inoculation produced a comparable response to inoculation with the control buffer. Single mutants of *P. chlororaphis* PCL1606 in *dar*B and *fit*D resulted in a slight decrease in mortality of approximately 15%, but they still had above 85% mortality in both cases. This drop in larval mortality shown by the PCL1606::*dar*B mutant (defective in HPR) recovered to wild-type levels when this mutation was complemented (strain ComB). A decrease of approximately 40% in mortality occurred when single and double mutants in *hcn*B and *prn*C genes were tested. However, differences were observed when *dar*B was involved in a double mutation with *hcn*B and *prn*C. Thus, in PCL1606 double mutants lacking HPR and PRN (PCL1606::*dar*B*prn*C) or HPR and HCN (PCL1606::*dar*B*hcn*B), the mortality levels observed increased to those shown by the wild-type strain values, which had higher mortality values than those displayed by the single mutant in *dar*B, *prn*C, or *hcn*B. A great difference was observed with the double mutant in the *dar*B and *fit*D genes (PCL1606::*dar*B*fit*D). The inoculation of this double mutant PCL1606::*dar*B*fit*D decreased larval mortality to non-inoculated control levels (Fig. 2).

### Involvement of HPR and Fit toxin in the toxicity of G. mellonella

**Fig. 3:**
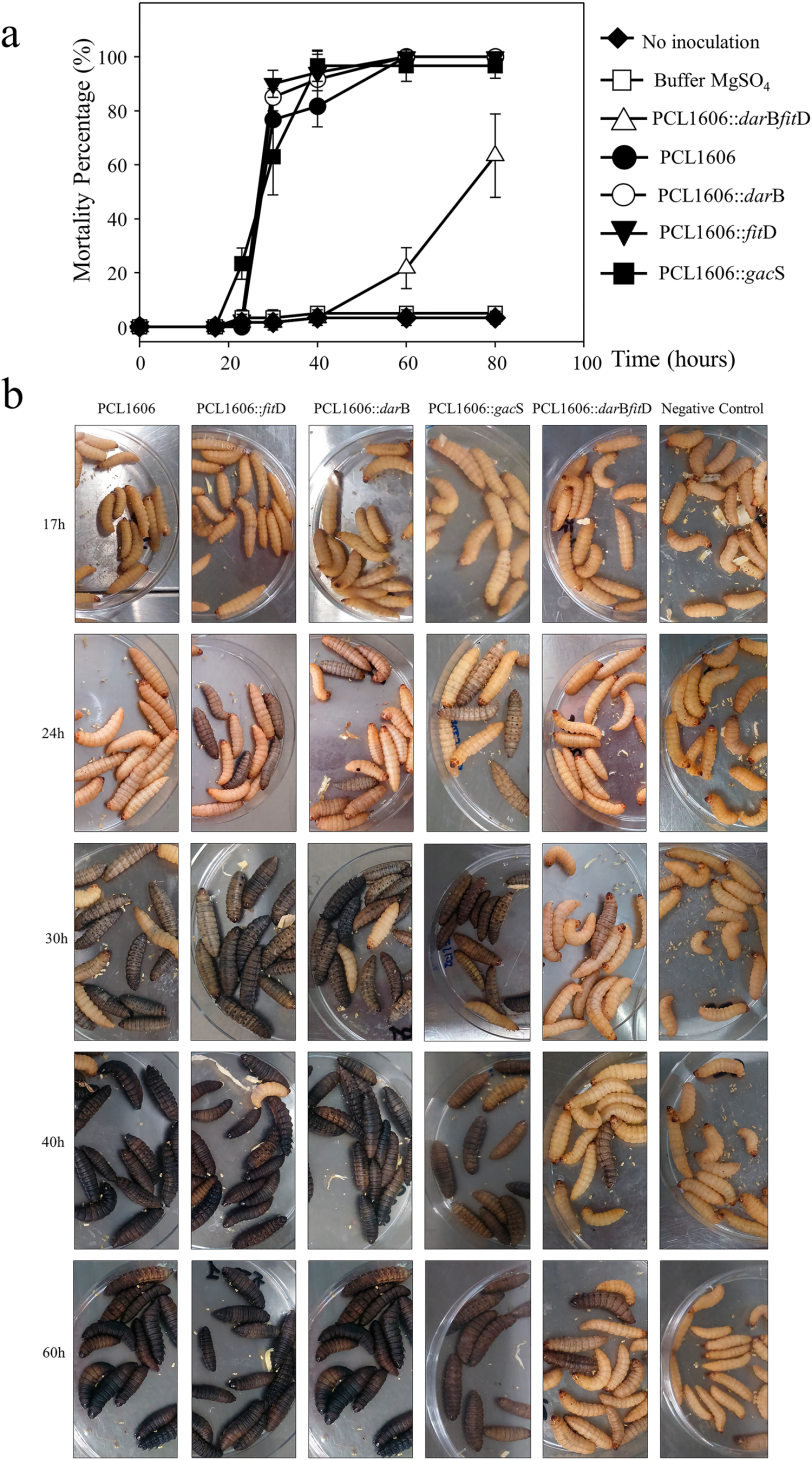
*Galleria mellonella* mortality assay, **a** percentage of mortality of *Galleria mellonella*, infected at dose 3 × 10^3^ cfu/mL along 80 h. Counting made at 0, 17, 24, 30, 40, 60, and 80-h post-inoculation. **b** Symptom’s development of *Galleria mellonella* infected at dose 3 × 10.^3^ cfu/mL incubated at 25 °C during 17, 24, 30, 40, and 60 h. Buffer inoculation was used as negative control. Healthy larva present beige color and black larvae represent the complete melanization and larvae death soon after

A mortality survey 80 h after inoculation with two different bacterial doses (3 × 10^3^ and 3 × 10^5^ cfu/mL; Fig. 3 and S1) was performed. The results showed that 40 h after inoculation, the wild-type *P. chlororaphis* PCL1606 and single mutants in *dar*B, *fit*D, and *gac*S retained the highest mortality; however, the double mutant PCL1606::*dar*B*fit*D reduced the *G. mellonella* mortality level to the buffer control values. This double mutant reached 60% *G. mellonella* larval mortality 80 h after inoculation. Therefore, there was a delay in the insecticidal activity of PCL1606 when HPR and Fit toxin were impaired in the same strain (Fig. 3a). With the higher dose, all events reached the highest mortality from 16 to 30 h after inoculation, but the double mutant HPR-Fit toxin showed a delay in killing the larvae, reaching 100% mortality 60 h after inoculation (Fig. S1).

The symptoms observed in *G. mellonella* larvae 24 h after the injection (3 × 10^3^ cfu/mL) with *fit*D- and *dar*B-defective mutants (Fig. 3b) showed that several larvae started to melanize. After 30 h, nearly all the larvae inoculated with the single mutants PCL1606::*fit*D, PCL1606::*dar*B, and PCL1606::*gac*S and the wild-type PCL1606 showed dark melanization. However, at 30-h postinoculation with buffer or with double mutant PCL1606::*dar*B*fit*D, the inoculated larvae did not display signs of melanization. 60 h after inoculation, they began to show symptoms of larval intoxication in those infected with the HPR-Fit toxin double mutant (Fig. 3b). The different mortality kinetics could be better distinguished at 30, 40, and 60 h after inoculation (Fig. S2, Fig. S3), showing that the wild-type PCL1606 strain presented progressively increasing mortality, reaching 100% at 60 h. The single mutants in *fit*D and *dar*B showed significantly higher virulence than the wild-type strain at 30 h. The single mutant in *gac*S seemed to be delayed until 40 h to display higher virulence. Finally, the double mutant seemed to be innocuous after 60 h, when insecticidal activity began (Fig. S2, Fig. S3).

## Discussion

*Pseudomonas chlororaphis* PCL1606 is a rhizobacterium isolated from avocado roots characterized by 2-hexyl-5-propyl resorcinol (HPR) production, the main compound involved in biocontrol (Cazorla et al. 2006), but also in other relevant phenotypes, such as biofilm formation and colonization (Calderón et al. 2019; Arrebola et al. 2019). A previous analysis of additional antifungal antibiotic compounds produced by PCL1606 revealed the production and presence of coding genes for hydrogen cyanide (HCN) and pyrrolnitrin (PRN). However, phenazine production and the presence of coding genes were not found in PCL1606 (Calderón et al. 2015). Additionally, the presence of genes homologous to the *fit*ABCDEFGH cluster, encoding a putative cytotoxin similar to Fit toxin, was detected in the genome of *P. chlororaphis* PCL1606 (Calderón et al. 2015). According to Ruffner et al. (2015), the Fit cytotoxin is restricted to a particular group of rhizobacteria comprised of *P. protegens* and *P. chlororaphis*, and it is strongly correlated with high insect toxicity.

In the current study, *G. mellonella* have been used as organism model to test the insecticidal capacity of *P. chlororaphis* PCL1606. *G. mellonella* is a pest insect of non-agricultural interest (since it lives in bee hives and not in plants like the insecticidal *Pseudomonas* sp.) and that the bacteria usually are taken orally in nature. Therefore, this study is only an approach for insecticidal activity, and in nature something else could happen. PCL1606 displayed insecticidal activity at the same level as the control strains *P. chlororaphis* subsp. *aurantiaca* (former *P. aurantiaca*) BL915 and *P. protegens* Pf-5, where HPR and Fit production were first described, respectively (Nowak-Thompson et al. 2003; Péchy-Tarr et al. 2008). On the other hand, the non-antagonistic rhizobacterium *P. alcaligenes* AVO110 did not show insecticidal capacity, did not have the *fit* or any antifungal antibiotic genes in its genome (Pintado et al. 2021), and did not produce antifungal secondary metabolites (Pliego et al. 2007; Pintado et al. 2021). The typical symptoms produced by the wild-type strain PCL1606 in *G. mellonella* larvae correspond to mortality accompanied by intense melanization from 24 h postinoculation. Melanization is a typical insecticidal symptom corresponding to the synthesis and deposition of melanin to encapsulate pathogens at the wound site followed by hemolymph coagulation and opsonization (Tsai et al. 2016).

Single and double mutants impaired the production of antifungal hydrogen cyanide (HCN) or pyrrolnitrin (PRN) compounds in PCL1606, resulting in a mortality reduction of *G. mellonella* larvae, but with no differences among them. This suggests that both compounds could only contribute to the insecticidal background and were not the main insecticidal compounds produced, as previously described for other insecticidal strains (Flury et al. 2017). It has been previously described that the insecticidal virulence of *P. protegens* CHA0 and *P. chlororaphis* PCL1391 mutants lacking one or several antibiotics, such as 2,4-diacetylphloroglucinol, phenazine, pyrrolnitrin, or pyoluteorin, were not reduced (Flury et al. 2017).

The role of HCN and PRN on insecticidal and nematicidal activity by antagonistic bacteria has been illustrated by other authors (Nandi et al. 2015; Kang et al. 2019), and our results indicate their involvement in the insecticidal phenotype displayed by the model strain PCL1606. In addition, this study included the PCL1606 derivative mutant defective in *gac*S, since *gac*S is a global regulator that can interfere with activities related to the production of secondary compounds, such as antifungal and insecticidal proteins (Saraf et al. 2011; Nandi et al. 2015; Flury et al. 2016, 2017). Previous studies have reported that mutants deficient in the global regulator *gac*S showed no insecticidal activity (Flury et al. 2017; Kang et al. 2019). These results do not agree with our observation where the impaired mutants in the *gac*S gene of PCL1606 still retain insecticidal activity, suggesting a *gac*S-regulated independent pathway responsible for the insecticidal phenotype. That insecticidal activity of *P. chlororaphis* PCL1606 could be regulated by different regulation system to gacA/gacS, or its regulation could be performed by gacA/gacS but supported by another system, such as type VI secretion system (T6SS) described by Vacheron et al. (2019) or two-partner secretion protein (TPS) as reported Vesga et al. (2020). The *P. chlororaphis* PCL1606 produce a resorcinol derivate (HPR) which antagonistic features have been studied (Cazorla et al. 2006; Calderón et al. 2014, 2015, 2019), but could have additional regulatory features (Brameyer et al. 2015). For this, the effects of HPR and Fit products were studied. Single mutants of these genes scarcely lowered *G. mellonella* larval mortality, but the absence of both genes completely impaired the insecticidal activity of the derivative bacteria, even in the presence of *hcn*B and *prn*C genes.

The insecticidal and nematicidal effectiveness of the Fit cytotoxin in *P. protegens* and *P. chlororaphis* has been documented (Kupferschmied et al. 2013; Péchy-Tarr et al. 2008). Heterologous expression of the Fit toxin in *Escherichia coli* resulted in the capacity of the bacterium to kill the insect host upon injection (Péchy-Tarr et al. 2008; Kupferschmied et al. 2013). In addition, the *fit*D defective mutants of *P. protegens* Pf-5 and CHA0 showed a decrease in insecticidal activity demonstrating that FitD makes an important contribution to insect virulence (Péchy-Tarr et al. 2008). However, FitD mutants who is not deficient in other toxins, such a as DAPG and/or HCN, showed limited insect toxicity, which seems to be related with bacterial cell, since injections of culture supernatant did not show larvae mortality (Péchy-Tarr et al. 2008). The fact that the *fit*D mutants still killed to 85% of *G. mellonella*, retained a certain level of toxicity, indicating to the authors that additional factors may contribute to anti-insect activity (Péchy-Tarr et al. 2008), in agreement with our observations on PCL1606. In the current study, the PCL1606 derivative mutant with the *fit*D toxin gene disrupted showed the capacity to kill *G. mellonella* at almost 80% 30 h post-infection. This result is not only due to HCN and PRN, as reported by the double derivative mutant in HPR and Fit toxin, with no virulence at this time post-infection. On the other hand, the double mutant HCN and PRN still showed that 60% of *G. mellonella* larvae died. Therefore, we can conclude that HCN and PRN could contribute to the insecticidal capacity of PCL1606, but they could be considered accompanying compounds. In fact, the virulence activity in *Pseudomonas* sp. has been general defined as a multifactorial trait (Rose et al. 2021; Vesga et al. 2021). Thus, the combination of HPR and the Fit toxin could be considered the main toxins responsible for insecticidal activity.

Focusing in the HPR and Fit toxin role in the insecticidal feature of *P. chlororaphis* PCL1606, it is observed that single derivative mutants in HPR and Fit toxin displayed the non-significant differences with wild-type in mortality levels of *G. mellonella*. However, the double derivative mutant PCL1606::*dar*B*fit*D was severely impaired in the phenotype, displaying a percentage of mortality close to negative control after approximately one day. HPR has been described as the main antibiotic against fungal pathogens such as *Rosellinia necatrix* or *Fusarium oxysporum* (Cazorla et al. 2006). However, HPR has been revealed as a versatile compound involved in bacterial adhesion, colony morphology and typical air–liquid interphase pellicles produced by PCL1606 (Calderón et al. 2019). Due to the chemical nature of HPR, it is possible that its involvement does not file because it is a component of the matrix but rather has regulatory connotations. There is evidence that resorcinol derivates could play a regulatory role in *Photorhabdus asymbiotica* (Brameyer et al. 2015), which extends its putative role in PCL1606. Interestingly, *P. asymbiotica* also has insecticidal capacity, thanks to the synthesis of Mcf proteins (Hapeshi and Waterfield 2016), where Mcf1 is similar molecule to FitD (Péchy-Tarr et al. 2008; Ruffner et al. 2015).

The combined antimicrobial and regulatory roles of HPR in PCL1606 could explain several results obtained in the present study. The toxic nature of HPR against different organisms (Kanda et al. 1975; Nowak-Thompson et al. 2003) could directly affect *G. mellonella* cells, as happens with antifungal phenazines (Wang et al. 2013), helping the insecticidal characteristics of *P. chlororaphis* PCL1606. This insecticidal activity would rival FitD in toxicity, thus justifying the single mutants PCL1606:*dar*B and PCL1606:*fit*D results in comparison with double mutant PCL1606::*dar*B*fit*D, also revealing that because of the absence of HPR and Fit toxin, there was no mortality due to HCN and PRN. On the other hand, HPR could have additional regulatory roles on secondary metabolites since alkylresorcinols can be involved as signaling molecules in a novel quorum sensing two-component regulatory system (Brameyer et al. 2015). Belonging to these sets of secondary metabolites could be chitinase and phospholipase C, putative genes that have been found in the PCL1606 genome (PCL1606_RS13585 as chitinase and PCL1606_RS14060 as phosphatase C). Both enzymes have also been reported to be important in insect pathogenicity (Flury et al. 2016). Beside these enzymes, other effectors have been reported that are involved in insecticidal activity of *Pseudomonas* sp. Vesga et al. (2020) showed evidence that TPS system (two-partner secretion system) could be part in the disruption of the epithelial cell after having passed through the peritrophic matrix. TPS of Gram-negative bacteria is formed by a B transporter and an A effector protein (Vesga et al. 2020). This type of secretion system has not been located in the genome of *P. chlororaphis* PCL1606. On the other hand, the type VI secretion system (T6SS) core apparatus and two VgrG modules contribute to the insect killing capacity in *P. protegens* CHA0 during oral infection but in injection assays (Vacheron et al. 2019). The genome annotation of *P. chlororaphis* PCL1606 have reveals the presence of T6SS component, as well as VgrG modules, which could participate in bacterial pathogenicity in case of oral infection. However, the current study has been focused on FitD involvement in insect toxicity of *P. chlororaphis* PCL1606, introducing the bacteria into the insect by injection, so the T6SS of PCL1606 should not have influenced the results, at least not decisively.

This also helps to explain the *gac*S mutant results, whose killing ability was not affected, suggesting a regulation of all the compounds at a higher hierarchy, but this regulatory role of HPR is still a hypothesis under study.

In summary from this first study, PCL1606 have the capacity to produce Fit toxin, a described compound with insecticidal capacity, and HPR, which has been shown to have insecticidal potential, to which its fungicidal character and its possible regulatory role must be added. This confirms HPR as one the main compounds produced by *P. chlororaphis* PCL1606 involved in the beneficial phenotypes displayed by this model bacterium.

## Supplementary Information

Below is the link to the electronic supplementary material.

Supplementary file1 (PDF 296 KB)

## Data Availability

The datasets generated during and/or analyzed during the current study are available from the corresponding author on reasonable request.
