# Supplementary material for: Insecticidal features displayed by the beneficial rhizobacterium Pseudomonas chlororaphis PCL1606
Source: Int Microbiol. 2022 Jun 7;25(4):679–89. doi: 10.1007/s10123-022-00253-w (PMC9526686; doi:10.1007/s10123-022-00253-w)
Supplement: Supplementary file 1 — Supplementary file1 (PDF 296 KB) [file 10123_2022_253_MOESM1_ESM.pdf]

**Electronic Supplementary Materials**

**《International Microbiology》**

# **Insecticidal features displayed by the beneficial rhizobacterium *Pseudomonas chlororaphis* PCL1606**

Eva Arrebola[1,2], Francesca R. Aprile [1,2], Claudia E. Calderón [1,2], Antonio de Vicente [1,2], Francisco M. Cazorla[1,2]

Corresponding author: Eva Arrebola. Mailing address: Departamento de Microbiología, Facultad de Ciencias, Universidad de Málaga, Boulevar Louis Pasteur nº 31, CP 29010, Málaga, Spain. E-mail: ead@uma.es

**Table S1** Percentage of mortality of *Galleria mellonella* in each of the symptom categories after 24h post-inoculation with each of the strains tested at a dose $10^5$ cfu/mL

| **Strains Tested** | **Symptom categories** | | | |
|---|---|---|---|---|
| | **0** | **1** | **2** | **3** |
| **Negative control** | | | | |
| No inoculation | 100 | | | |
| $MgSO_4$ buffer | 97 | | 3 | |
| **Wildtype bacterias control** | | | | |
| *Pseudomonas psedualcaligenes* AVO110 | | 100 | | |
| *Pseudomonas aurantiaca* BL915 | | | | 100 |
| *Pseudomonas protegens* Pf5 | | | | 100 |
| *Pseudomonas chlororaphis* PCL1606 | | 5 | 95 | |
| **Derivatives antibiotics mutants of PcPCL1606** | | | | |
| **HPR mutants** | | | | |
| PCL1606::darB | | 15 | 85 | |
| ComB | | | 100 | |
| **Pyrrolnitrin mutant** | | | | |
| PCL1606::prnC | | 40 | 60 | |
| **Hydrogen cyanide mutant** | | | | |
| PCL1606::hcnB | | 40 | 60 | |
| **Citotoxin** | | | | |
| PCL1606::fitD | | 10 | 90 | |
| **Double mutants** | | | | |
| PCL1606::darBprnC | | | 100 | |
| PCL1606::darBhcnB | | | 80 | 20 |
| PCL1606::prnChcnB | | 40 | 60 | |
| PCL1606::darBfitD | 100 | | | |
| **Derivative regulating mutants of PcPCL1606** | | | | |
| PCL1606::gacS | | | 13 | 87 |

**Symptom categories**

**0** **1** **2** **3**

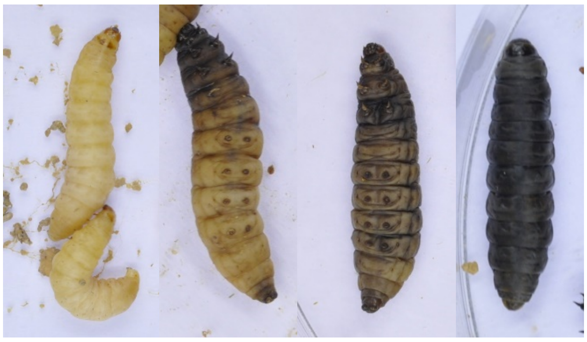

**Figure S1**

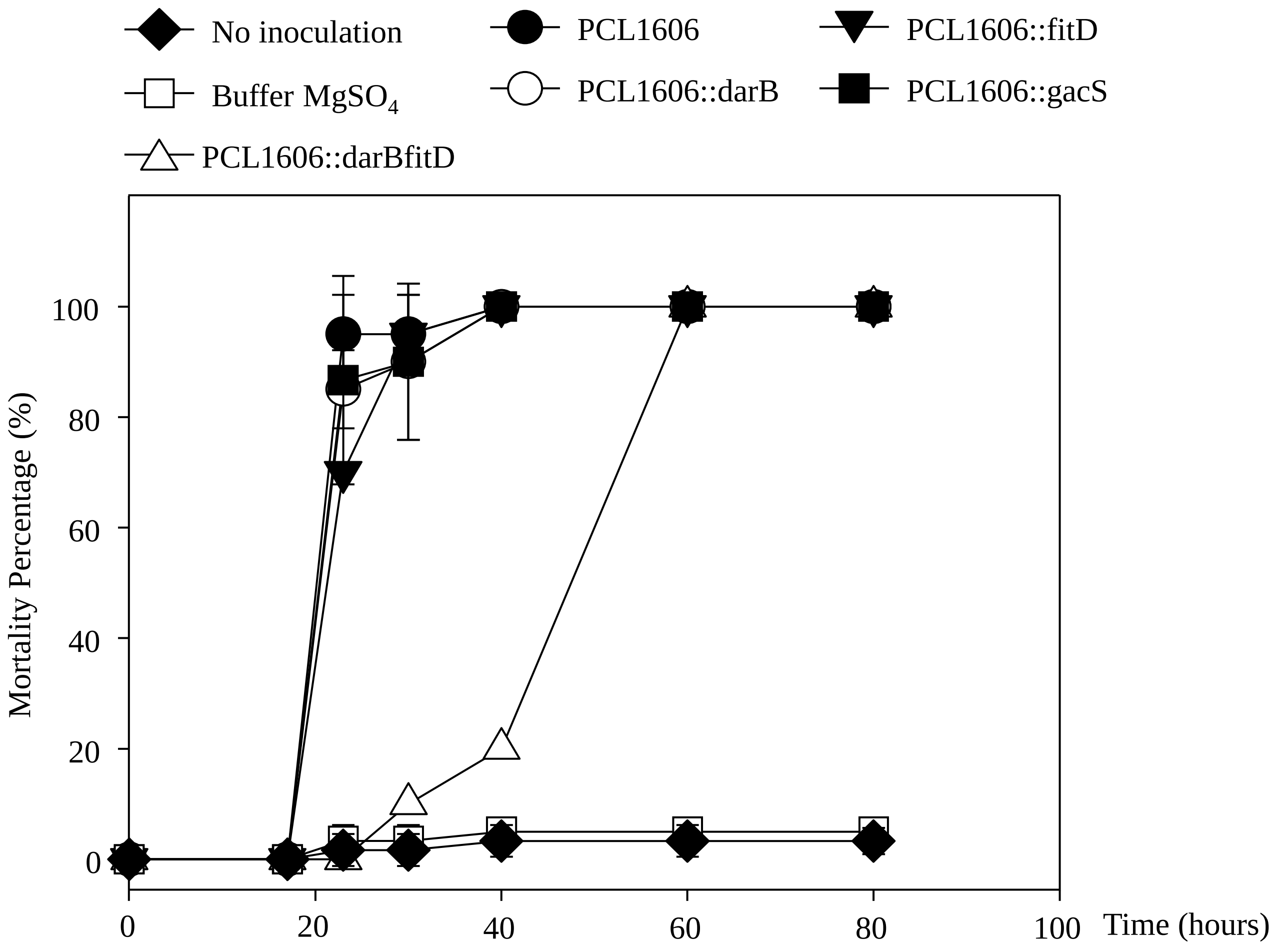


**Fig. S1** Percentage of mortality of *Galleria mellonella*, infected at dose $3x10^5$ cfu/mL along eighty hours. Counting made at 0, 17, 24, 30, 40, 60 and 80-hours post-inoculation

**Figure S2**

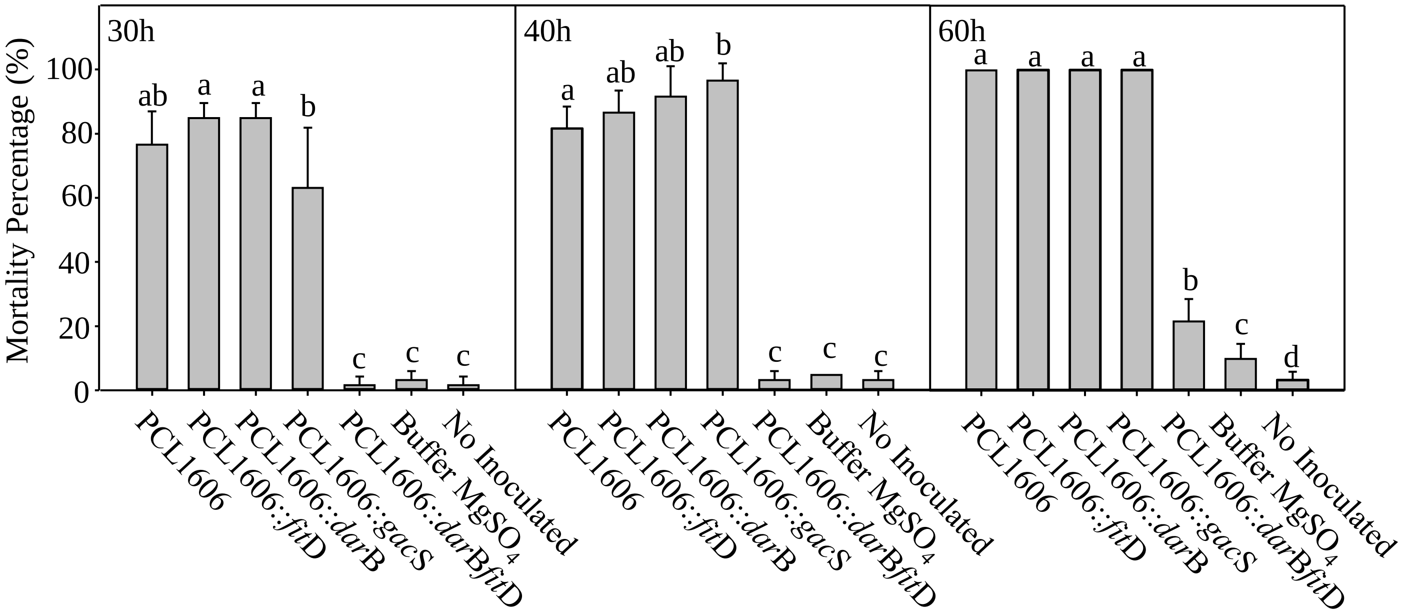


**Fig. S2** Mortality of *Galleria mellonella* represented by percentage of dead larvae at 30, 40 and 60 hours of incubation post-infection at dose $3x10^3$ cfu/mL. Statistical analysis by ANOVA was performed using IBM SPSS 22 software (SPSS Inc., Chicago, IL, United States).

**Figure S3**

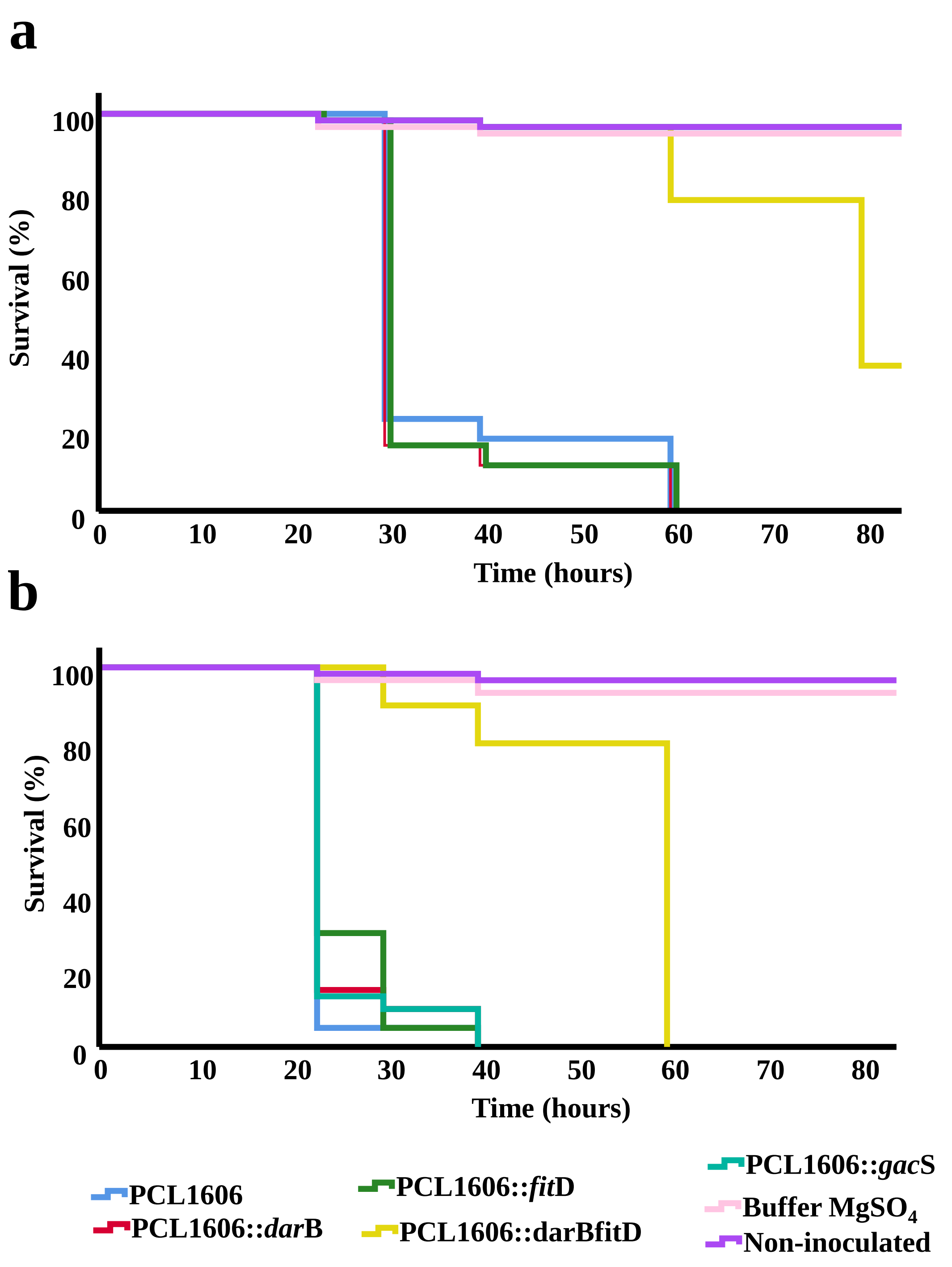


**Fig. S3** Kaplan-Meier survival curves of *Galleria mellonella* larvae infected with the wild-type *Pseudomonas chlororaphis* PCL1606 and diferent derivative strains. Larvae (n = 20, per group) were injected with a) $3 \times 10^3$ or b) $3{\times}10^5$ cfu/mL per tested bacteria. Control groups consisted of larvae treated with a 10 µl dose of buffer $MgSO_4$ (10 mM). Survival was monitored every 12-24 h over a period of 80 h. Data are pooled from a minimum of three independent experiments. Injection with the wild-type and derivative single mutant strains resulted in significantly higher mortality rate compared to injection with the control strains and with the double mutant in *fitD* and *darB genes* ($P<0.05$, log rank test).

Survival data were plotted using the Kaplan–Meier method and comparisons between groups were made using the log-rank test using IBM SPSS statistics v 25
